# Papillary thyroid microcarcinoma with synchronous asymptomatic advanced esophageal squamous cell carcinoma: A case report and review of the literature

**DOI:** 10.3892/ol.2014.2748

**Published:** 2014-12-01

**Authors:** PU CHENG, YINGYING XIANG, ENDONG CHEN, ZHANGYONG ZOU, XIAOHUA ZHANG

**Affiliations:** Department of Surgical Oncology, The First Affiliated Hospital of Wenzhou Medical University, Wenzhou, Zhejiang 325000, P.R. China

**Keywords:** synchronous, thyroid carcinoma, esophageal squamous cell carcinoma, rare case

## Abstract

Papillary thyroid microcarcinoma (PTMC) is a subset of papillary thyroid carcinoma, with tumors measuring ≤1.0 cm in maximum diameter. Esophageal squamous cell carcinoma (ESCC) is the most common histopathological subtype of the various esophageal malignancies. The present study reports a rare synchronous presentation of PTMC and asymptomatic ESCC manifesting as lateral cervical lymph node enlargement. The patient, a 70-year-old male who presented to the Department of Surgical Oncology, The First Affiliated Hospital of Wenzhou Medical University (Wenzhou, China)with no significant symptoms, was identified to have bilateral thyroid nodules by ultrasonography and enlarged lymph nodes on the left side of the neck by palpation. A diagnosis of PTMC with lateral cervical lymph node metastasis was established and the patient underwent right lobectomy, partial left lobectomy and lymph node biopsy. Pathological analysis of the intraoperative frozen biopsy indicated bilateral PTMC and metastatic lymph node SCC from a different primary location. Furthermore, histological examination of the biopsied lymph nodes revealed bilateral PTMC and poorly-differentiated SCC, which was verified as ESCC by gastroscopy and postoperative histopathological examination. A review of the present literature indicated that no similar case has been reported thus far. The patient succumbed three months after attending Ruijin Hospital, Shanghai Jiao Tong University School of Medicine (Shanghai, China) for additional treatment.

## Introduction

Papillary thyroid microcarcinoma (PTMC) is a subset of papillary thyroid carcinoma, with tumors measuring ≤1.0 cm in maximum diameter. Clinically, PTMC is frequently accompanied by lymph node metastasis, however, the mortality rate is relatively low (1,2).

Esophageal squamous cell carcinoma (ESCC) is the most common histopathological subtype of the various esophageal malignancies. It is among the most aggressive types of cancer and is characterized by early-stage lymph node metastasis as well as a high rate of mortality (3,4). Furthermore, ESCC patients are occasionally asymptomatic, despite having reached the metastatic stage, at the time of initial diagnosis.

Synchronous primary malignancies refer to the occurrence of a novel type of cancer concurrently or within six months of the diagnosis of the initial primary malignancy (5). The present study conducted a literature review, which indicated that no report currently exists documenting a simultaneous case of PTMC and ESCC with no clinical manifestation, with the exception of lateral cervical lymphadenopathy. However, the present report describes such a case in a 70-year-old male. Written informed consent was obtained from the patient’s family.

## Case report

**Figure 1:**
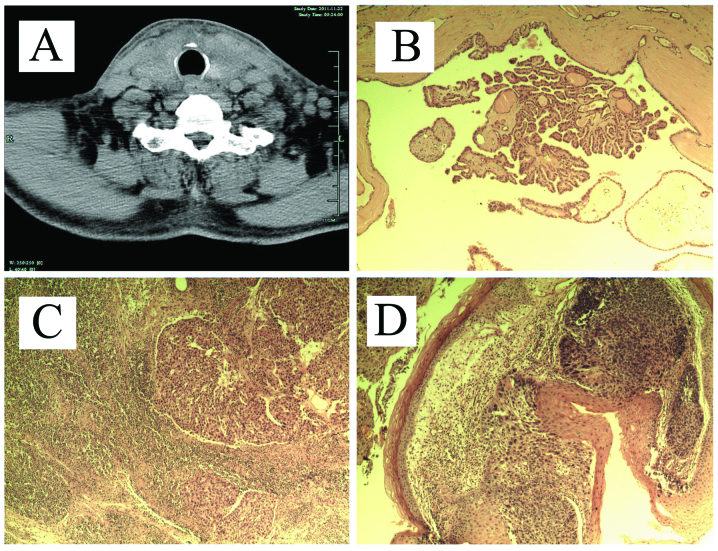
(A) Multiple enlarged lymph nodes were detected via postoperative computed tomography. (B) Histological examination of the resected specimen confirmed the diagnosis of papillary thyroid microcarcinoma (magnification, ×40; staining, H&E). (C) The histological type of the lymph node lesion was poorly-differentiated squamous cell carcinoma (magnification, ×40; staining, H&E). (D) Pathology reports of samples obtained upon gastroscopy verified esophageal squamous cell carcinoma (magnification, ×40; staining, H&E). H&E, hematoxylin and eosin.

A 70-year-old male was admitted to the Department of Surgical Oncology (The First Affiliated Hospital of Wenzhou Medical University, Wenzhou, China) in November, 2011 with suspected PTMC and lateral cervical lymph node metastasis identified by careful digital palpation and ultrasonography (US) during a routine health examination. The patient had a >2-year medical history of hypertension, which was controlled with antihypertensive agents (indapamide and amlodipine besylate) and a 10-year history of smoking half a pack of cigarettes per day. There was no remarkable family history and the patient was in good general health with no significant weight loss. The thyroid gland appeared normal upon physical examination and no abnormalities were identified on the chest X-ray, electrocardiogram or pulmonary function examination. Furthermore, laboratory examinations, including thyroid hormone and tumor marker analysis, were unremarkable. Enlarged lymph nodes on the left neck were painless, solid and immobile, and could be identified by palpation; the largest lymph node measured 2.0 cm in diameter. Ultrasonographic examination identified one hyperechoic nodular lesion ~0.4 cm in diameter in the lower pole of left lobe, and three well-circumscribed, heterogeneous hypoechoic nodular lesions with a diameter of ~0.9 cm in the lower pole of the right lobe, ~1.0 cm in the superior pole and ~0.4 cm in the lower pole of left lobe. No obvious indicators of metastasis were identified. In consideration of the abovementioned data, the patient was diagnosed with thyroid carcinoma and consequently underwent a right lobectomy, a partial left lobectomy and a lymph node biopsy. Analysis of the intraoperative frozen pathological sample revealed a diagnosis of bilateral PTMC with metastatic lymph node SCC from a different primary location. Furthermore, all of the thyroid tumors were surrounded by an intact capsule and demonstrated no definitive evidence of capsular extension or invasion, however, the location of the primary lesion was unclear, thus, the surgery was prematurely terminated. Multiple enlarged lymph nodes were detected via postoperative computed tomography (CT), particularly above the clavicle (Fig. 1A). The patient recovered well with no major complications and was discharged after six postoperative days. Subsequent histopathological examination confirmed the diagnosis of papillary thyroid microcarcinoma without extrathyroidal extension in the two lobes (Fig. 1B) and the histological type of the lymph node lesion was determined to be poorly-differentiated SCC (Fig. 1C). One week after the surgery was performed, the patient returned for additional examination. Gastroscopy was performed on the recommendation of the present authors; this procedure indicated the presence of ESCC and pathological analysis verified this diagnosis (Fig. 1C). The patient succumbed three months after attending Ruijin Hospital, Shanghai Jiao Tong University School of Medicine (Shanghai, China) for radiotherapy.

## Discussion

In addition to the trends of an aging population and the development of novel diagnostic techniques, the incidence rate of single patients exhibiting multiple primary malignant neoplasms has seen a rapid global rise. However, the occurrence of co-existing primary cancers involving the esophagus and thyroid gland remains rare. The present study conducted a review of the literature regarding this condition and determined that there have been a total of just 10 published cases (5–10). However, the majority of these studies were not published in English and certain cases contain insufficient detail. Table I summarizes the diagnosis and prognosis data for all of the 11 cases previously reported, including the current patient. To the best of our knowledge, the present study reports the first case of asymptomatic, synchronous development of PTMC and ESCC.

In the majority of cases, patients diagnosed with occult thyroid malignancies do not exhibit any clinical symptoms during their lives. The use of thyroid US and US-guided fine-needle aspiration biopsy (FNAB) has made it easier to detect PTMC. Although PTMC is always benign itself, thus, resulting in a favorable clinical outcome, cases accompanied by regional lymph node and distant metastasis have been well documented in the literature (11–13). In the current patient, PTMC was considered to be the most likely diagnosis, based on the imaging features of asymptomatic nodules identified by US and the enlarged lymph nodes identified by palpation of the left neck. Management of thyroid lesion included the performance of a right lobectomy, partial left lobectomy and lymph node biopsy. Analysis of the frozen pathological sample indicated a diagnosis of poorly differentiated SCC, which was in accordance with the previous diagnosis of bilateral PTMC. As the primary lesion of the metastatic lymph nodes could not be identified, surgery was terminated. Pathological examination confirmed the prior diagnosis of PTMC and subsequent analysis determined synchronous ESCC. The current patient succumbed due to extensive ESCC metastasis during additional treatment three months following discharge. Of note, the present study highlighted that, in patients with an asymptomatic thyroid carcinoma, cervical lymphadenopathy does not always indicate metastatic disease. Furthermore, the presence of different histopathological in primary thyroid lesions and suspicious lymph nodes indicates the possibility of synchronous malignancies.

More than half a million incidences of SCC of the head and neck are reported worldwide per year (14), with a high incidence of lymph node metastasis and poor prognosis (14,15). Clinically, lymph node metastasis from asymptomatic primary SCC is not uncommon, however, it remains difficult to identify the primary lesion due to the lack of efficient auxiliary examination. Primary SCC of the thyroid is rare with a high mortality rate (16,17), therefore, it was not considered in the initial diagnosis. Instead, ESCC was considered; ESCC occurs relatively frequently, constituting 7% of all gastrointestinal cancers. The typical symptoms of primary ESCC are progressive dysphagia and pain behind the sternum, while stenosis of the esophagus or thickening of the esophageal wall can be determined by performing US, CT and other imaging techniques. Occasionally, patients observed to exhibit no apparent clinical symptoms upon diagnosis are already in an advanced stage of the disease. In the present case, the patient was advised to undergo gastroscopy and positron emission tomography-CT if necessary to identify the primary lesion. Although pathological analysis confirmed the diagnosis of ESCC, the optimum opportunity for treatment had already passed; therefore, the patient succumbed to the disease.

Synchronous malignancies pose multiple difficulties, diagnostically for the pathologist and in terms of management for the clinician. At present, the rate of misdiagnosis for synchronous primary ESCC and thyroid carcinomas continues to be high (18,19). Although additional diagnostic procedures, such as CT and US, are frequently used, they rarely improve the accuracy of the diagnosis. For example, when cervical nodules are identified using US or CT in patients with suspected primary thyroid carcinoma, it is difficult to distinguish metastasis from discrete primary disease. Therefore, surgeons should be aware of the possibility of the tumor representing metastatic disease from primary growth at a distinct site, for example the esophagus, during the treatment process (20). However, if a definitive diagnosis is established, the preferred method for treating the two malignancies is surgery. Improving preoperative diagnosis may make simultaneous removal of the two lesions possible and contribute to a longer survival time for patients with synchronous primary carcinomas. Thus, there is a clear requirement for evaluating properties of the cervical masses to improve diagnosis.

In conclusion, the present study recommends that, in cases of asymptomatic synchronous development of PTMC and ESCC, FNAB should be conducted prior to surgery to aid in the development of the treatment strategy. If a cervical metastatic lymph node of SCC is identified, the clinician should consider the possibility of synchronous ESCC, in order to reduce the rate of misdiagnosis. To achieve the optimum therapeutic efficacy and prognosis, the present study proposes that adjuvant preoperative examinations, such as US and CT, and pathologic examinations by endoscopic excision biopsy may be valuable. Diagnosis should not rely on experience alone; instead all options should be considered, resulting in earlier treatment and improved outcomes.

## Figures and Tables

**Table I:** Cases of synchronous primary cancer involving the esophagus and thyroid gland.

				Cancer type		
					
Reporters	Year	Age, years	Gender	Esophageal (stage)	Thyroid	Prognosis, months (status)
Naomoto *et al* (5)	1990	46	Female	SCC (IV)	Fol.	20 (succumbed)
	1990	56	Female	SCC (IV)	Pap.	22, (succumbed)
	1991	74	Male	SCC(IV)	Fol.	10 (succumbed)
	1993	55	Female	SCC (IV)	Pap.	14 (alive)
Hamanaka *et al* (6)	1975	67	Female	SCC (IV)	Pap.	12 (succumbed)
	1975	69	Female	Unknown	Fol.	12 (succumbed)
Takeshita *et al* (7)	1978	59	Male	SCC (IV)	Fol.	19 (alive)
Yoshinaka *et al* (8)	1980	58	Male	SCC (IV)	Fol.	2 (succumbed)
Nishida *et al* (9)	1988	54	Female	SCC (IV)	Pap.	14 (alive)
Juhász A *et al* (10)	2005	55	Male	Unknown	Pap.	Unknown (alive)
Present case	2011	70	Male	SCC (IV)	Pap.	3 (succumbed)

SCC, squamous cell carcinoma; Pap., papillary carcinoma; Fol., follicular carcinoma.

## References

[1] Besic N, Pilko G, Petric C, Hocevar M, Zgajnar J (2008). Papillary thyroid microcarcinoma: prognostic factors and treatment. J Surg Oncol.

[2] Giordano D, Gradoni P, Oretti G, Molina E, Ferri T (2010). Treatment and prognostic factors of papillary thyroid microcarcinoma. Clin Otolaryngol.

[3] Wang GQ, Abnet CC, Shen Q (2005). Histological precursors of oesophageal squamous cell carcinoma: results from a 13 year prospective follow up study in a high risk population. Gut.

[4] Fukuhara T, Hiyama T, Tanaka S, Oka S, Yoshihara M, Arihiro K, Chayama K (2010). Characteristics of esophageal squamous cell carcinomas and lugol-voiding lesions in patients with head and neck squamous cell carcinoma. J Clin Gastroenterol.

[5] Naomoto Y, Haisa M, Yamatsuji T (1999). Multiple primary cancers of the esophagus and thyroid gland. Jpn J Clin Oncol.

[6] Hamanaka Y, Narita N, Tsuboi K, Onoda K, Ose D, Takemura H (1975). Two cases with concurrent esophageal cancer and thyroid cancer. Geka Shinryo.

[7] Takesita T, Tokugawa H, Kurihara M (1978). A case of esophageal cancer with thyroid cancer. Gan No Rinsho.

[8] Yoshinaka H, Suenaga T, Tanabe H (1981). Two cases of double cancer of esophagus and thyroid. Rinsho Geka.

[9] Nishida Y, Nagamachi Y, Tanaka M (1988). Studies of esophageal cancer and cancers of other organs. Geka.

[10] Juhász A, Szilágyi A, Elso I, Tihanyi Z, Paál B, Altorjay A (2005). Synchronous carcinoma of the esophagus and the thyroid gland. Orv Hetil.

[11] Baudin E, Travagli JP, Ropers J (1998). Microcarcinoma of the thyroid gland: the Gustave Roussy Institute experience. Cancer.

[12] Ardito G, Revelli L, Giustozzi E (2013). Aggressive papillary thyroid microcarcinoma: prognostic factors and therapeutic strategy. Clin Nucl Med.

[13] Anastasilakis AD, Polyzos SA, Makras P (2012). Papillary thyroid microcarcinoma presenting as lymph node metastasis - a diagnostic challenge: case report and systematic review of literature. Hormones (Athens).

[14] Haddad RI, Shin DM (2008). Recent advances in head and neck cancer. N Engl J Med.

[15] Seiwert TY, Cohen EE (2005). State-of-the-art management of locally advanced head and neck cancer. Br J Cancer.

[16] Makay O, Kaya T, Ertan Y (2008). Primary squamous cell carcinoma of the thyroid: report of three cases. Endocr J.

[17] Zimmer PW, Wilson D, Bell N (2003). Primary squamous cell carcinoma of the thyroid gland. Mil Med.

[18] Ponnie TTP, Simon YKL, Chu K-M, Frank JB, John W (1997). Multiple primary cancers in esophageal squamous cell carcinoma: incidence and implications. Ann Thorac Surg.

[19] Shibuya H, Takagi M, Horiuchi J, Suzuki S, Kamiyama R (1982). Carcinomas of the esophagus with synchronous or metachronous primary carcinoma in other organs. Acta Radiol Oncol.

[20] Abemayor E, Moore DM, Hanson DG (1988). Identification of synchronous esophageal tumors in patients with head and neck cancer. J Surg Oncol.

